# Association of automated quantified emphysema and interstitial lung abnormality with survival in non-small cell lung cancer

**DOI:** 10.1186/s13244-025-02180-6

**Published:** 2026-01-05

**Authors:** Guangjing Weng, Junli Tao, Yu Pu, Changyu Liang, Bohui Chen, Zhenyu Wang, Chengzhan Qi, Jiuquan Zhang

**Affiliations:** 1 School of Medicine, Chongqing University, Chongqing University Cancer Hospital, Chongqing, China; 2 Department of Radiology, Chongqing University Cancer Hospital & Chongqing Cancer Institute, Chongqing, China

**Keywords:** AI-quantified, Emphysema, Interstitial lung abnormality, Non-small cell lung cancer, Prognosis

## Abstract

**Objectives:**

To investigate the prognostic value of artificial intelligence (AI) quantified emphysema and interstitial lung abnormality (ILA) in patients with non-small cell lung cancer (NSCLC).

**Materials and methods:**

This retrospective study used AI to quantify emphysema and ILA in patients diagnosed with NSCLC between January 2015 and December 2017. Associations between AI-quantified emphysema and ILA severity and overall survival (OS) were evaluated using Cox proportional hazards models. The ability of AI-quantified emphysema and ILA severity to predict OS was explored via concordance index (C-index) and area under the time-dependent receiver operating characteristic curve (AUC). Furthermore, exploratory OS analyses were performed on subgroups stratified by chronic obstructive pulmonary disease status, treatment type, and tumor-node-metastasis (TNM) staging.

**Results:**

Of 1675 patients, 830 (49.6%) survived, and 845 (50.4%) died. Whole emphysema (mild: HR, 1.66 [95% CI: 1.26, 2.18]; *p* < 0.001; more than mild: HR, 2.55 [95% CI: 1.88, 3.48]; *p* < 0.001) and ILA (equivocal ILA: HR, 1.63 [95% CI: 1.15, 2.32]; *p* = 0.006; definite ILA: HR, 2.33 [95% CI: 1.61, 3.35]; *p* < 0.001) severity were independent prognostic factors for NSCLC, while regional emphysema and regional ILA severity were not. The model combining AI-quantified whole emphysema severity and ILA severity outperformed the TNM staging-only model in predicting NSCLC patient outcome (C-index, 0.80 vs. 0.75; AUC, 0.90 vs. 0.85).

**Conclusions:**

Increased AI-quantified whole emphysema and ILA severity were associated with worse OS in NSCLC. The model combining AI-quantified emphysema and ILA showed improved performance for predicting patient survival versus TNM staging alone.

**Critical relevance statement:**

AI-quantified emphysema and ILA severity are associated with NSCLC patient outcome and can provide information complementary to TNM staging for predicting NSCLC patient survival and promoting the development of individualized management strategies.

**Key Points:**

- The study explores artificial intelligence (AI) quantified emphysema and interstitial lung abnormality (ILA) severity in non-small cell lung cancer (NSCLC) prognosis.
- The AI-quantified whole emphysema severity and ILA severity were independent prognostic factors for NSCLC patient outcome, while regional emphysema and regional ILA severity were not.
- AI-quantified emphysema and ILA severity may help predict the survival of NSCLC patients and help clinicians make informed treatment decisions.

**Graphical Abstract:**

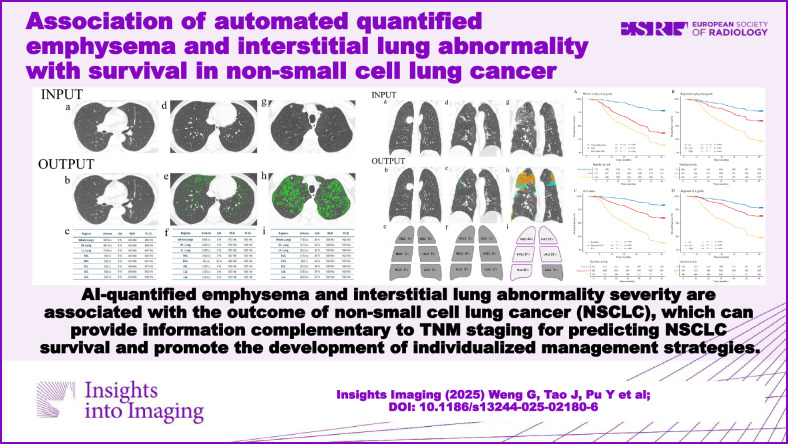

## Introduction

Approximately 85% of lung cancer cases are non-small cell lung cancer (NSCLC), with a 5-year overall survival (OS) rate of only 25% [1]. The prognostic stratification of NSCLC primarily relies on tumor-node-metastasis (TNM) staging [2]. However, TNM staging is not an optimal prognostic tool for NSCLC due to the clinical heterogeneity of patients at the same stage [3]. Therefore, it is important to investigate additional indicators to improve NSCLC prognosis stratification.

Comorbidities are associated with NSCLC survival, with chronic obstructive pulmonary disease (COPD) representing the most common lung cancer comorbidity [4]. A recent study reported that COPD was correlated with worse OS rate in NSCLC patients [5]. Pulmonary function test (PFT) is the gold standard for diagnosing COPD. However, 10–95% of patients with lung function impairment have normal PFT, suggesting that PFT potentially leads to the underdiagnosis of COPD [6, 7]. Additionally, PFT depends on patient cooperation, and some lung cancer patients cannot tolerate the test [8]. Thus, it is necessary to develop an accurate and acceptable approach for providing information complementary to PFT.

Emphysema is recognized as the typical phenotype of COPD [9]. The latest Global Initiative for Chronic Obstructive Lung Disease report indicated that interstitial lung abnormality (ILA) is closely associated with COPD [10]. So, emphysema and ILA may be potential markers of COPD. Chest CT is regarded as the optimal non-invasive method for assessing emphysema and ILA [11, 12]. Previous studies have shown that NSCLC without emphysema and ILA per CT visual assessment had a favorable outcome [13, 14]. Furthermore, more severe emphysema and ILA are associated with worse prognoses [15, 16]. The outcome of NSCLC patients with severe regional emphysema has also been found to be poorer than that of patients with mild and moderate [17]. However, visual subjective evaluation is time-consuming, has poor reproducibility and requires dedicated training [12]. As artificial intelligence (AI) advances in medical imaging, AI-quantified emphysema and ILA are being explored to overcome these limitations [18, 19].

To our knowledge, this is the first study to evaluate the prognostic value of AI-quantified emphysema and ILA in NSCLC survival. The purpose of this study was to assess whether AI-quantified emphysema and ILA are associated with NSCLC prognosis. Furthermore, we investigated whether AI-quantified emphysema and ILA severity improve OS prediction, independent of TNM staging alone. Additionally, exploratory OS analyses were conducted in subgroups stratified by COPD status, treatment type, and TNM staging.

## Materials and methods

This study was approved by the institutional review boards of our hospital and waived the requirement for written informed consent (CZLS2022109-A).

### Study sample and follow-up

We collected baseline data from patients diagnosed with NSCLC between January 2015 and December 2017. The data consisted of demographics (age and sex), clinical data (smoking history, family history, hypertension, diabetes mellitus, coronary artery calcification and treatment type), serum tumor markers (carcinoembryonic antigen and carbohydrate antigen 125), pathological data (TNM staging and histologic subtype), and radiological data (tumor size and location were documented according to the baseline radiological report). We also analyzed PFT parameters measured within 2 weeks of the chest CT, including forced expiratory volume in the first second (FEV₁), forced vital capacity (FVC), diffusing capacity of the lung for carbon monoxide (DLCO), and the FEV₁/FVC ratio. A postbronchodilator FEV1/FVC < 0.70 was used to confirm the diagnosis of COPD [20].

The inclusion criteria were as follows: *(a)* histopathologically confirmed NSCLC; *(b)* baseline chest thin-section CT images (≤ 1.25 mm) available on our Picture Archiving and Communication System; *(c)* complete baseline data; and *(d)* no previous clinical suspicion of interstitial lung disease. The exclusion criteria were *(a)* presence of other primary malignancies; *(b)* poor image quality that was inadequate for evaluation; and *(c)* missing follow-up or inability to obtain follow-up outcome. This study’s outcome was the 5-year OS rate, defined as the time from diagnosis to the date of death from any cause or the date of the last follow-up.

### Chest CT acquisition

Chest CT scans were performed utilizing the 64-multidetector Philips Brilliance CT. The Supplementary Materials provide a detailed chest CT scan and settings parameters.

### Emphysema and ILA visual assessment

Visual assessment of baseline chest CT images was used to determine the presence of whole emphysema and ILA and their degrees of severity. Visual assessment criteria are detailed in the Supplementary Materials.

### AI-quantified emphysema and ILA

AI quantification software [19, 21, 22] (A-VIEW, version 1.1.46.11, Suhai Alderi Information Technology Ltd.) was used to analyze emphysema and ILA in baseline chest CT images. Firstly, we uploaded the CT images into the software. Then, the AI automatically quantified the ratio of whole emphysema and further segmented the lobar (right upper, right middle, right lower, left upper, and left lower) to quantify the regional emphysema ratio. Regarding ILA analysis, the lung parenchyma was segmented into six zones according to anatomical landmarks (levels of the inferior aortic arch and right inferior pulmonary vein), and the AI automatically quantified the ILA ratios in these zones [11]. We obtained the following parameters: whole emphysema ratio, regional emphysema ratio, and ILA ratio in each lung zone.

The participants were divided into three categories according to the whole emphysema ratio: (1) non-emphysema; (2) mild (less than 5%); and (3) more than mild (more than 5%) [23, 24]. Furthermore, the patients were categorized according to the regional emphysema ratio into the no, low (emphysema ratio < 1% in the lobar with tumor) and high (emphysema ratio ≥ 1% in the lobar with tumor) groups [25]. Regarding ILA severity, the participants were classified into three categories: (1) non-ILA; (2) equivocal ILA (the presence of any nondependent changes affecting < 5% of any lung zone or with unilateral changes); and (3) definite ILA (the presence of any nondependent changes affecting ≥ 5% of any lung zone) [11, 26, 27]. As the regional ILA severity criteria have not been confirmed, the optimal cutoff value of regional ILA severity was determined using X-tile software (version 3.6.1; Yale University School of Medicine) as previously described [3, 28–30]. The patients were subsequently divided into three categories according to regional ILA ratio: no, low (ILA ratio < 1.2% in the lung zone with tumor), and high (ILA ratio ≥ 1.2% in the lung zone with tumor).

**Fig. 1:**
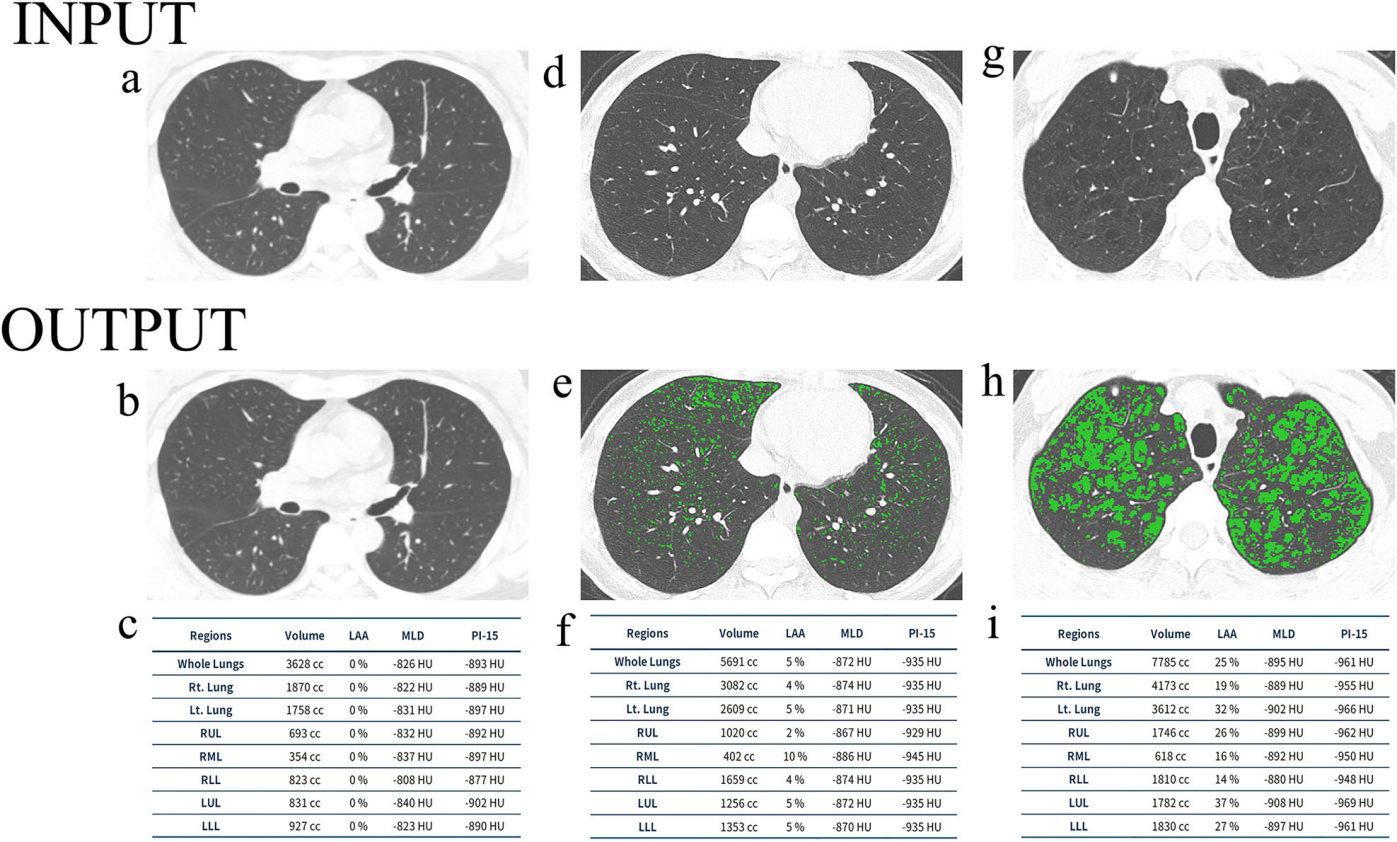
Example outputs for artificial intelligence (AI) quantified emphysema. A threshold of −950 HU was used to define emphysema. The AI-quantified outputs label the CT images to indicate the severity of emphysema, and a quantification table of emphysema is also provided. Axial CT images in participants with non-emphysema (**a**–**c**), mild emphysema (**d**–**f**), and more than mild emphysema (**g**–**i**). Individuals with mild emphysema and more than mild emphysema exhibit increased CT emphysema voxels (green areas) versus those without emphysema

**Fig. 2:**
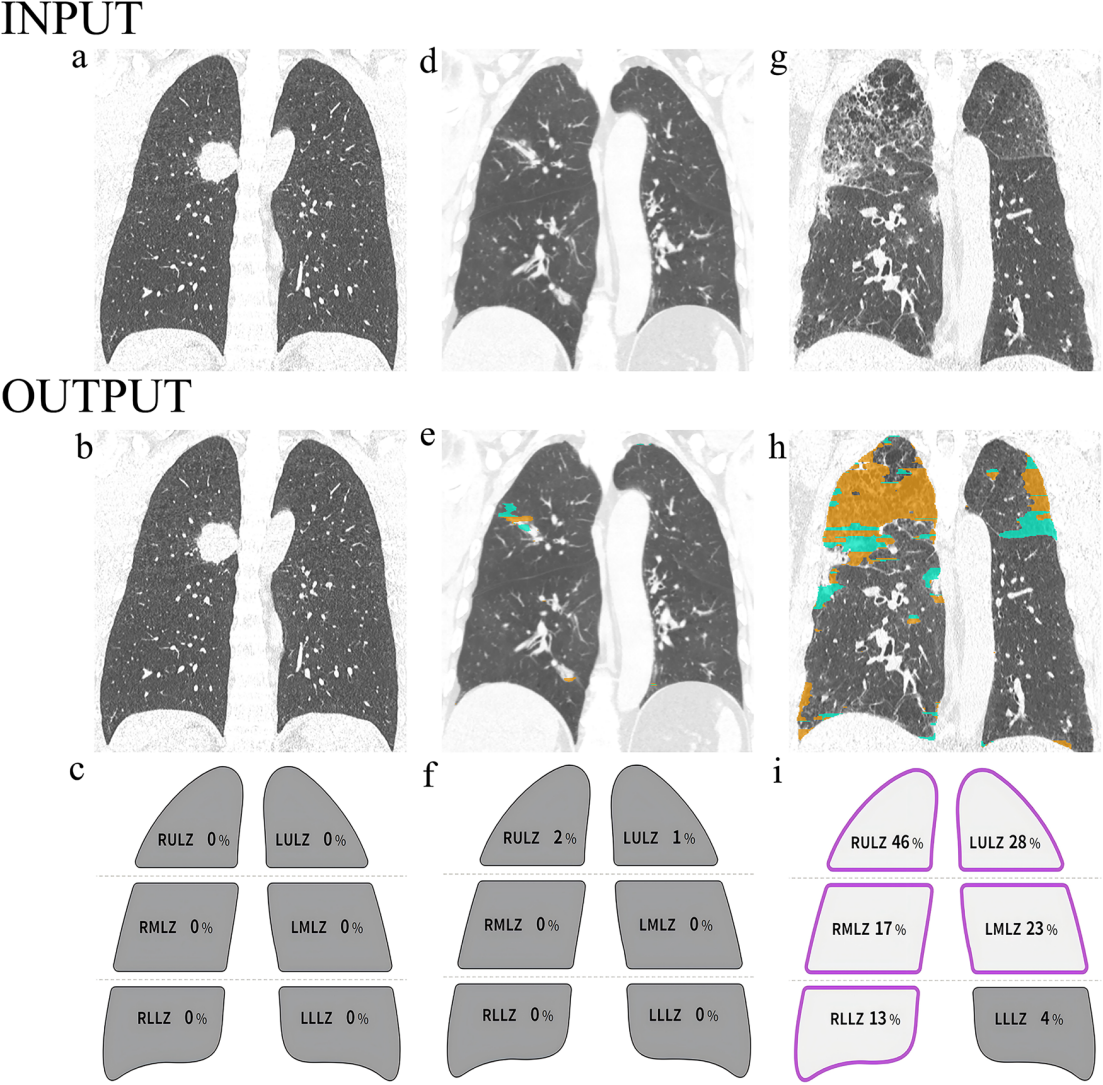
Example outputs for artificial intelligence (AI) quantified interstitial lung abnormality (ILA). For ILA measurement, the lung parenchyma was segmented into six lung zones, demarcated by the upper, middle, and lower lung zones, which are defined by the levels of the inferior aortic arch and the right inferior pulmonary vein. The AI-quantified outputs label the CT images to indicate ILA severity and provide a quantification schematic diagram of ILA. Coronal CT images in participants with non-ILA (**a**–**c**), equivocal ILA (**d**–**f**), and definite ILA (**g**–**i**). Equivocal ILA and definite ILA exhibit increased green and yellow areas versus those observed without ILA

The schematic diagrams of AI-quantified emphysema and ILA were presented in Figs. 1 and 2, respectively.

### Survival analysis

Kaplan–Meier curves were produced and compared using the log-rank test. Univariable and multivariable Cox regression analysis were used to estimate hazard ratio (HR) and corresponding 95% confidence intervals (CIs). The multiplicative interaction between emphysema and ILA was determined using a likelihood ratio test to compare the Cox models with and without the emphysema and ILA of the interaction term. In addition, additive interactions were assessed using the relative excess risk due to interaction and the attributable proportion of the interaction that was obtained from the Cox model with the interaction term.

### Sensitivity analysis

To evaluate the reliability of the optimal threshold for regional ILA severity determined by X-tile, three sets of sensitivity analyses were undertaken: (1) we reanalyzed the association between regional ILA severity and 5-year OS by excluding participants whose death occurred within the first years of follow-up in order to minimize reverse causality; (2) we considered that the OS of NSCLC differed according to age, sex, smoking history and tumor size, and therefore stratified participants according to age (< 65 years, ≥ 65 years), sex, smoking history and tumor size (< 3 cm, ≥ 3 cm); and (3) we used the multiple imputations were used for the imputation of covariates to assess whether the excluded patients with missing covariate values would influence our results [31].

### Added value of AI-quantified emphysema and ILA over TNM staging alone

We constructed 7 prognostic predictive models per the multivariable analysis: Model 1: TNM staging; Model 2: whole emphysema severity; Model 3: ILA severity; Model 4: TNM staging and whole emphysema severity; Model 5: TNM staging and ILA severity; Model 6: whole emphysema severity and ILA severity; Model 7: TNM staging, whole emphysema severity and ILA severity.

Time-dependent receiver operating characteristic (ROC) curves were used to evaluate the performance of the models. The DeLong test was applied to compare the areas under the ROC curves (AUC) for 5-year OS between the best-performing model and the others. The concordance index (C-index) and Akaike Information Criterion (AIC) assessed model goodness-of-fit, while calibration curves with the Hosmer-Lemeshow test were applied to evaluate the agreement between predicted and observed risks and to assess model robustness. Clinical efficacy was evaluated by decision curve analysis. Shapley additive explanations plots were utilized to visualize the contribution of Model 7 selected factors to OS.

### Relationship between AI-quantified metrics and surgical resection by stages I–IIIA

We explored the AI-quantified emphysema and ILA’s potential impact on eligibility for surgery in patients with resectable NSCLC (stages I–IIIA). Logistic regression analysis was fitted for the odds ratio (OR) of surgical resection by AI-quantified emphysema and ILA. We distinguish between stage IA and stages IB–IIIA because there is no indication for adjuvant treatment after surgery for stage IA, while adjuvant therapy is considered for stages IB–IIIA [5].

### Subgroup analysis

Participants were stratified by: (1) COPD status (non-COPD and COPD groups); (2) treatment type (non-surgery and surgery groups); and (3) TNM staging criteria (stages I–IIIA and stages IIIB–IV groups) [13, 32, 33]. The Supplementary Materials provide detailed subgroup analysis.

### Statistical analysis

Details of the variables analysis, the agreement and correlation analysis between the observer and AI were provided in the Supplementary Materials. The visual analysis of emphysema severity was classified into 5 categories by the Fleischner Society. However, due to the limited number of patients with trace, confluent and advanced destructive emphysema, we divided the patients with emphysema into 2 categories (mild and more than mild) to maintain sufficient statistical power [34]. All statistical analyses were performed by using SPSS Statistics (version 27.0; IBM Corp.) and R software (version 4.0.0; R Foundation for Statistical Computing). All *p*-values were two-sided, and *p* < 0.05 was considered to indicate significance.

## Results

### Participant characteristics

**Fig. 3:**
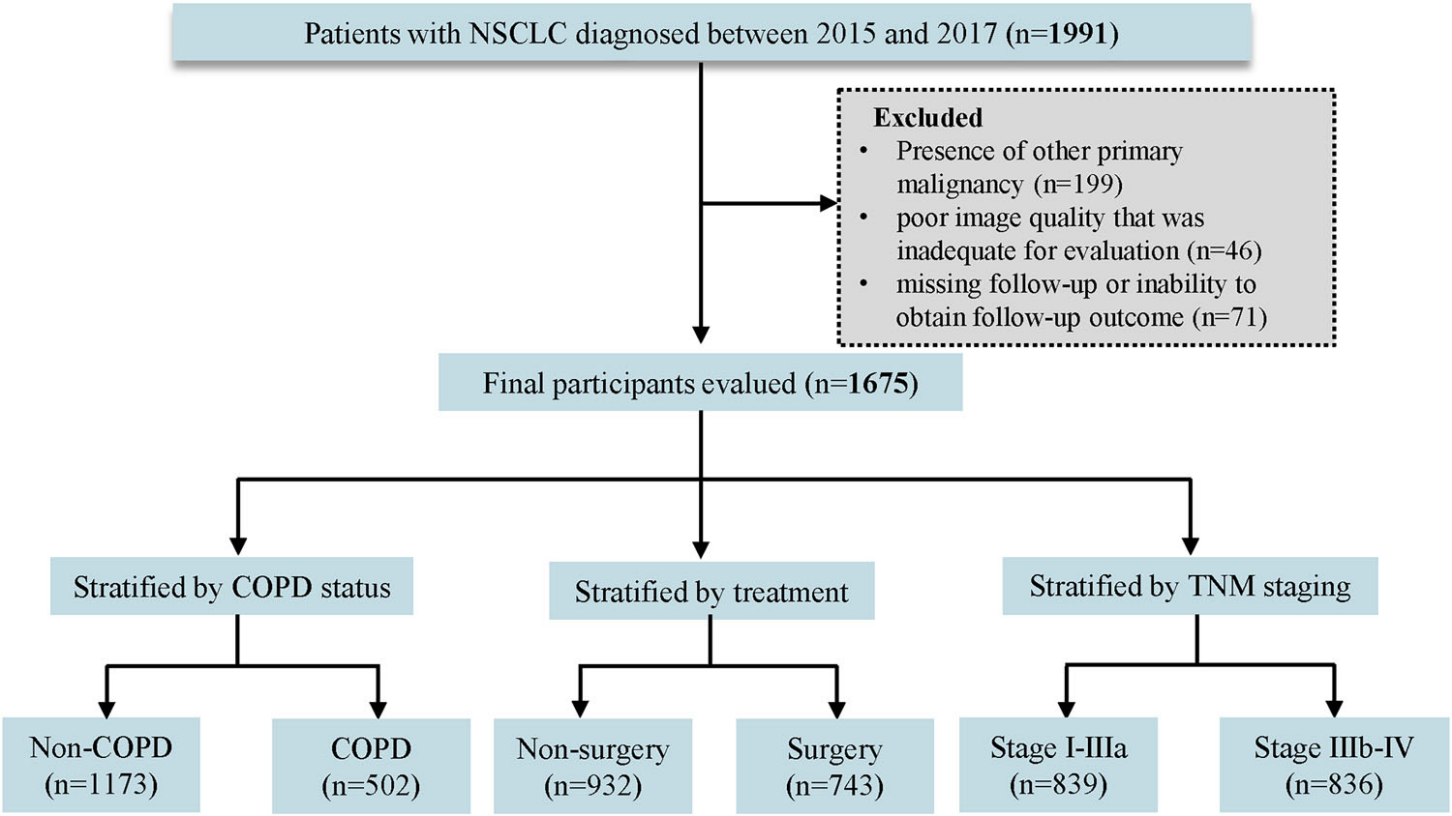
Flowchart showing study sample selection and inclusion. COPD, chronic obstructive pulmonary disease; TNM, tumor-node-metastasis

The original cohort comprised 1991 participants, excluding 316 participants, 1675 were ultimately included (Fig. 3). Of the 1675 patients, 830 (49.6%) survived and 845 (50.4%) died. The median age (interquartile range) of the survival group was 57 (50–64) years, and 49.2% (*n* = 480) of the patients were female. Among the participants who died, 513 were men (60.7%) and 332 were women (39.3%); the median age was 62 (54–68) years. The baseline characteristics are shown in Table 1.

**Table 1:** Baseline characteristics on the basis of mortality

Parameter	Survival (*n* = 830)	Death (*n* = 845)	*p*-value
Age (years)	56.02 ± 11.46	60.08 ± 9.71	< 0.001
Sex			< 0.001
Male	422 (50.8)	513 (60.7)	
Female	408 (49.2)	332 (39.3)	
Smoking history			< 0.001
No	519 (62.5)	446 (52.8)	
Yes	311 (37.5)	399 (47.2)	
Family history			0.01
No	794 (95.7)	784 (92.8)	
Yes	36 (4.3)	61 (7.2)	
Hypertension			0.01
No	725 (87.3)	698 (82.6)	
Yes	105 (12.7)	147 (17.4)	
Diabetes mellitus			0.78
No	747 (90.0)	764 (90.4)	
Yes	83 (10.0)	81 (9.6)	
Coronary artery calcification			0.07
No	766 (92.3)	758 (89.7)	
Yes	64 (7.7)	87 (10.3)	
CEA (ng/mL)			< 0.001
Normal (≤ 5)	701 (84.5)	533 (63.1)	
Increased (> 5)	129 (15.5)	312 (36.9)	
CA125 (U/mL)			< 0.001
Normal (≤15)	663 (79.9)	500 (59.2)	
Increased (> 15)	167 (20.1)	345 (40.8)	
Tumor size (cm)			< 0.001
≤ 3	505 (60.8)	305 (36.1)	
> 3	325 (39.2)	540 (63.9)	
Tumor location			< 0.001
Peripheral	682 (82.2)	548 (65.1)	
Central	148 (17.8)	294 (34.9)	
TNM staging			< 0.001
I	476 (57.3)	62 (7.3)	
II	183 (22.0)	37 (4.4)	
III	75 (9.0)	265 (31.4)	
IV	96 (11.6)	481 (56.9)	
Treatment			< 0.001
Non-surgery	324 (39.0)	608 (72.0)	
Surgery	506 (61.0)	237 (28.0)	
Histologic type			< 0.001
Adenocarcinoma	668 (80.5)	547 (64.7)	
Squamous cell carcinoma	139 (16.7)	268 (31.7)	
Other NSCLC	23 (2.8)	30 (3.6)	
Pulmonary function test			
FVC (L)	3.09 ± 1.80	2.70 ± 0.63	< 0.001
FEV1 (L)	2.38 ± 0.53	2.01 ± 0.51	< 0.001
FEV1/FVC (%)	78.63 ± 9.54	73.22 ± 10.12	< 0.001
DLCO (L)	18.76 ± 3.65	17.03 ± 3.14	< 0.001
COPD status			< 0.001
Non-COPD	703 (84.7)	470 (55.6)	
COPD	127 (15.3)	375 (44.4)	
Whole emphysema severity			< 0.001
Non-emphysema	614 (74.0)	178 (21.1)	
Mild	155 (18.7)	272 (32.2)	
More than mild	61 (7.3)	395 (46.7)	
Whole emphysema ratio (%)	0.18 (0–0.57)	4.62 (1.39–6.85)	< 0.001
Regional emphysema severity			< 0.001
No	376 (45.3)	113 (13.4)	
Low	319 (38.4)	225 (26.6)	
High	135 (16.3)	507 (60.0)	
Regional emphysema ratio (%)	0.10 (0–0.49)	1.11 (0.33–3.32)	< 0.001
ILA severity			< 0.001
Non-ILA	359 (43.3)	74 (8.8)	
Equivocal ILA	402 (48.4)	281 (33.2)	
Definite ILA	69 (8.3)	490 (58.0)	
ILA ratio (%)	0.12 (0.01–0.60)	5.21 (3.77–6.49)	< 0.001
Regional ILA severity			< 0.001
No	335 (40.4)	94 (11.1)	
Low	336 (40.5)	204 (24.1)	
High	159 (19.2)	547 (64.7)	
Regional ILA ratio (%)	0.15 (0.01–0.87)	2.09 (1.00–5.72)	< 0.001

Unless otherwise indicated, numbers represent the count of patients, with percentages in parentheses. Whole emphysema ratio, regional emphysema ratio, ILA ratio and regional ILA ratio were reported as median and interquartile range. TNM staging was based on the 9th AJCC system for lung cancer

*p* < 0.05 indicates a statistically significant difference

*CEA* carcinoembryonic antigen, *CA125* carbohydrate antigen 125, *TNM* tumor-node-metastasis, *FVC* forced vital capacity, *FEV1* forced expiratory volume in 1 s, *DLCO* diffusing capacity of lung for carbon monoxide, *COPD* chronic obstructive pulmonary disease, *NSCLC* non-small cell lung cancer

### Emphysema and ILA assessment agreement

The Kappa agreement between the observer and AI for the presence of whole emphysema, whole emphysema severity, the presence of ILA and ILA severity was excellent, with κ values of 0.87, 0.87, 0.83 and 0.85, respectively. The correlation coefficients between the AI and visual assessment for the presence and severity of whole emphysema were 0.87 and 0.93, respectively. Those for the presence of ILA and ILA severity were 0.83 and 0.92, respectively.

### Survival analysis

**Fig. 4:**
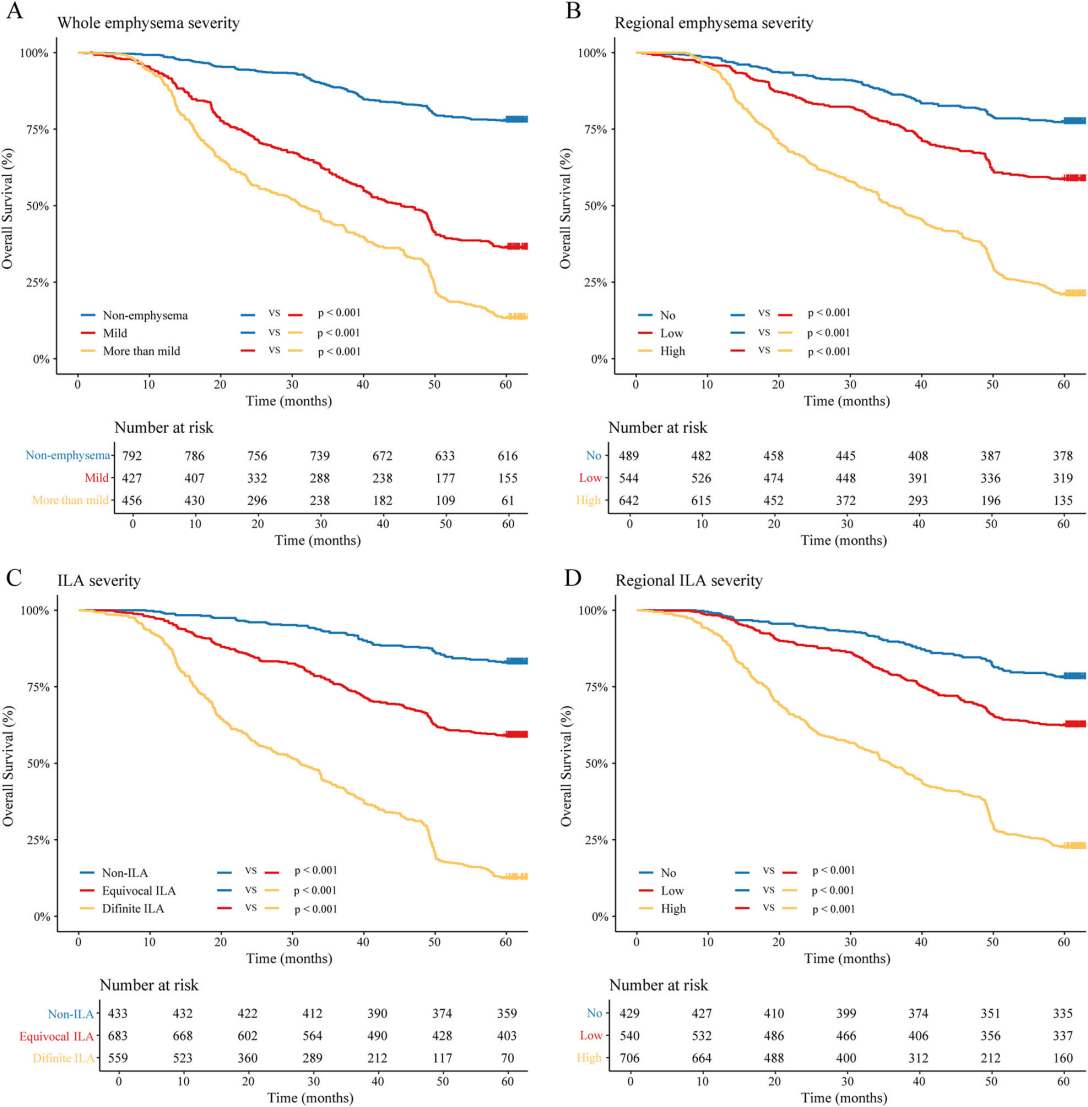
Kaplan–Meier curves showing NSCLC patient 5-year overall survival rate. Whole emphysema severity (**A**), regional emphysema severity (**B**), ILA severity (**C**), and regional ILA severity (**D**). ILA, interstitial lung abnormality. *p* < 0.05 was considered to indicate significance

The Kaplan–Meier survival curves indicated that patients with emphysema, whether whole or regional, had worse survival than those without emphysema (Fig. 4A, B). The OS of patients with mild emphysema was better than that of patients with more than mild emphysema (*p* < 0.001), and patients with high regional emphysema had lower OS rates than those with low regional emphysema (*p* < 0.001). Similarly, patients without ILA had a favorable prognosis (Fig. 4C, D). Survival rates decreased as ILA severity and the extent of regional ILA increased (both *p* < 0.001).

Multivariable analysis demonstrated that whole emphysema severity (mild: HR, 1.66 [95% CI: 1.26, 2.18]; *p* < 0.001; and more than mild: HR, 2.55 [95% CI: 1.88, 3.48]; *p* < 0.001), and ILA severity (equivocal ILA: HR 1.63 [95% CI: 1.15, 2.32]; *p* = 0.006; and definite ILA: HR 2.33 [95% CI: 1.61, 3.35]; *p* < 0.001) were the independent prognostic factors for NSCLC, while regional emphysema and regional ILA severity (both *p* > 0.05) were not (Table 2). A multiplicative interaction was observed between emphysema and ILA for the 5-year OS rate (HR 5.65 [95% CI: 4.84, 6.59]; *p* < 0.001). However, no additive interaction was observed (relative excess risk due to interaction, *p* = 0.07; and attributable proportion of interaction, *p* = 0.07) (Table 3).

**Table 2:** Univariable and multivariable Cox regression analyses of clinicopathological characteristics, CT-defined emphysema and interstitial lung abnormality severity for overall survival

Variables	Univariable analysis HR (95% CI)	*p*-value	Multivariable analysis HR (95% CI)	*p*-value
Age (years)	1.03 (1.03, 1.04)	< 0.001	1.02 (1.01, 1.02)	< 0.001
Sex (Male)				
Female	0.70 (0.61, 0.81)	< 0.001	1.16 (0.96, 1.40)	0.12
Smoking history (No)				
Yes	1.44 (1.25, 1.64)	< 0.001	1.09 (0.92, 1.30)	0.32
Family history (No)				
Yes	1.55 (1.19, 2.01)	0.001	1.267 (0.97, 1.65)	0.08
Hypertension (No)				
Yes	1.32 (1.11, 1.58)	0.002	0.86 (0.71, 1.04)	0.11
Diabetes mellitus (No)				
Yes	0.95 (0.75, 1.19)	0.64		
Coronary artery calcification (No)				
Yes	1.12 (0.90, 1.41)	0.29		
CEA (ng/mL) (≤ 5)				
Increased (> 5)	2.18 (1.90, 2.51)	< 0.001	0.86 (0.73, 1.01)	0.06
CA125 (U/mL) (≤ 15)				
Increased (> 15)	2.22 (1.94, 2.55)	< 0.001	1.12 (0.96, 1.31)	0.15
Tumor size (cm) (≤ 3)				
> 3	2.07 (1.80, 2.38)	< 0.001	1.08 (0.93, 1.27)	0.32
TNM staging (stage I)				
II	1.48 (0.99, 2.23)	0.06	0.86 (0.56, 1.30)	0.47
III	12.32 (9.33, 16.27)	< 0.001	5.06 (3.70, 6.93)	< 0.001
IV	15.44 (11.83, 20.15)	< 0.001	6.49 (4.81, 8.76)	< 0.001
Treatment (Non-surgery)				
Surgery	0.35 (0.30, 0.41)	< 0.001	0.64 (0.54, 0.75)	< 0.001
Histologic type (Adenocarcinoma)				
Squamous cell carcinoma	1.82 (1.57, 2.11)	< 0.001	0.99 (0.83, 1.19)	0.93
Other NSCLC	1.33 (0.92, 1.92)	0.13	0.86 (0.59, 1.27)	0.46
Tumor location (Peripheral)				
Central	1.86 (1.61, 2.14)	< 0.001	1.10 (0.93, 1.29)	0.28
COPD status (Non-COPD)				
COPD	2.75 (2.40, 3.16)	< 0.001	1.10 (0.95, 1.29)	0.22
Whole emphysema severity (Non-emphysema)				
Mild	4.08 (3.37, 4.93)	< 0.001	1.66 (1.26, 2.18)	< 0.001
More than mild	7.30 (6.10, 8.73)	< 0.001	2.55 (1.88, 3.48)	< 0.001
Regional emphysema severity (No)				
Low	2.04 (1.63, 2.56)	< 0.001	0.98 (0.74, 1.30)	0.90
High	5.60 (4.56, 6.88)	< 0.001	0.73 (0.53, 1.02)	0.06
ILA severity (Non-ILA)				
Equivocal ILA	2.91 (2.25, 3.75)	< 0.001	1.63 (1.15, 2.32)	0.006
Definite ILA	10.47 (8.18, 13.40)	< 0.001	2.33 (1.61, 3.35)	< 0.001
Regional ILA severity (No)				
Low	1.94 (1.52, 2.48)	< 0.001	0.94 (0.69, 1.30)	0.72
High	6.02 (4.83, 7.50)	< 0.001	1.31 (0.95, 1.80)	0.10

Data in parentheses are 95% CI. TNM staging was based on the 9th AJCC system for lung cancer

*p* < 0.05 indicates a statistically significant difference

*CEA* carcinoembryonic antigen, *CA125* carbohydrate antigen 125, *TNM* tumor-node-metastasis, *COPD* chronic obstructive pulmonary disease, *ILA* interstitial lung abnormality, *NSCLC* non-small cell lung cancer, *HR* hazard ratio

**Table 3:** Interaction between CT-defined emphysema and interstitial lung abnormality on 5-year overall survival

	HR	RERI	AP
Model	5.65	10.02	0.48
*p*-value	< 0.001	0.07	0.07

Multiplicative interaction is assessed using HR. RERI and AP indicate additive interaction

*p* < 0.05 indicates a statistically significant difference

*ILA* interstitial lung abnormality, *RERI* relative excess risk due to interaction, *AP* attributable proportion, *HR* hazard ratio

### Sensitivity analysis

Supplementary Table S1 presents the results of sensitivity analyses excluding participants who died within the first year of follow-up, showing that regional ILA severity was not an independent predictor of OS (*p* > 0.05). Stratified analyses by age, sex, smoking history, and tumor size also showed no significant associations (all *p* > 0.05; Supplementary Table S1). Additionally, results remained materially unchanged after multiple imputation of covariates (*p* > 0.05; Supplementary Table S1). In summary, the sensitivity analyses were consistent with the primary findings, indicating that regional ILA severity was not an independent prognostic factor for NSCLC. Therefore, it was excluded from model construction and is unlikely to influence the predictive performance of the model.

### Incremental prognostic value of emphysema and ILA

**Fig. 5:**
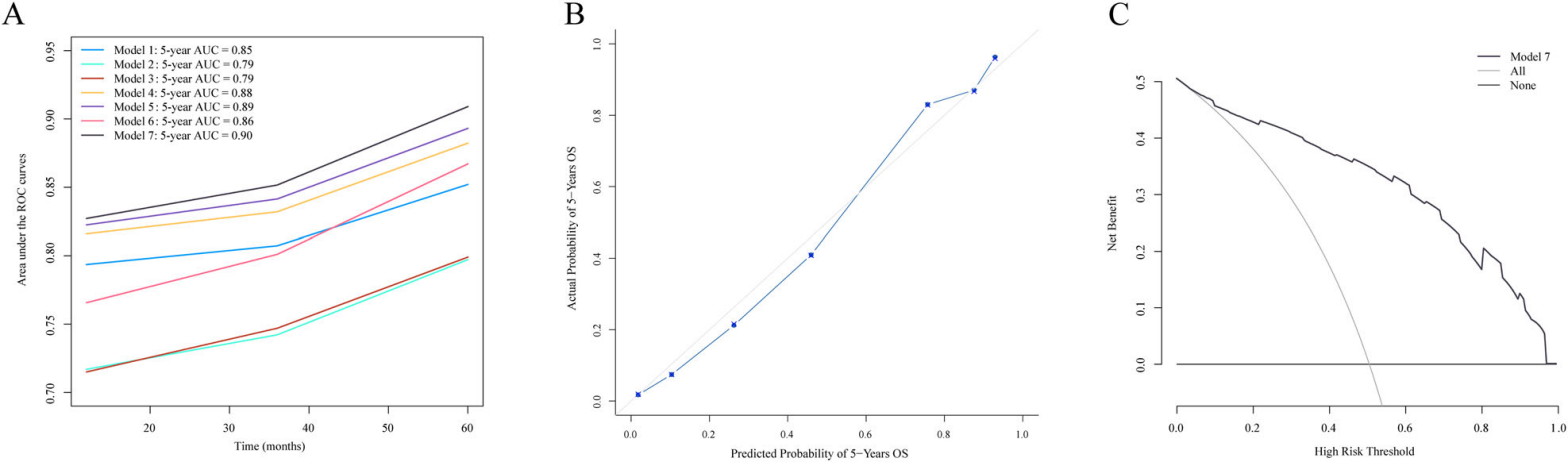
Prognostic performance and clinical usefulness of models. **A** Time-dependent areas under the ROC curve of the models. **B** The calibration curve of Model 7 shows the agreement between predicted and observed 5-year survival rate. The Hosmer-Lemeshow goodness-of-fit test shows that the *p*-value for model 7 is greater than 0.05. **C** The decision curve analysis of Model 7. The *y*-axis measures the net benefit, while the *x*-axis represents the different probability thresholds of mortality risk. The gray line represents the assumption that all patients have died by the fifth year. The black line represents the assumption that no patients have died by the fifth year. The modeling factors are all independent prognostic factors. Model 1: TNM staging. Model 2: whole emphysema severity. Model 3: ILA severity. Model 4: TNM staging and whole emphysema severity. Model 5: TNM staging and ILA severity. Model 6: whole emphysema severity and ILA severity. Model 7: TNM staging, whole emphysema severity and ILA severity. ROC, the time-dependent receiver operating characteristic curve; ILA, interstitial lung abnormality; TNM, tumor-node-metastasis

Model 7, incorporating TNM staging, whole emphysema and ILA severity, achieved superior predictive performance for the 5-year OS (AUC: 0.90; Table 4), significantly outperforming other models (DeLong test: all *p* < 0.001). It also showed the highest C-index (0.80) and the lowest AIC (719.02), indicating good model fit and robustness. Time-dependent ROC further confirmed the incremental prognostic value of AI-quantified emphysema and ILA severity (Fig. 5A). The model demonstrated excellent agreement between predicted and observed outcome (Fig. 5B) and showed favorable clinical utility (Fig. 5C). The performance of Models 1–6 is detailed in the Supplementary Materials.

**Table 4:** Additional predictive value of AI-quantified whole emphysema severity and interstitial lung abnormality severity in combination with TNM staging

	AIC	Concordance index	5-year AUC	*p*-value
Model 1	810.54	0.76 (0.75–0.77)	0.85	< 0.001
Model 2	848.64	0.71 (0.70–0.72)	0.79	< 0.001
Model 3	842.81	0.71 (0.71–0.72)	0.79	< 0.001
Model 4	800.36	0.78 (0.78–0.79)	0.88	< 0.001
Model 5	793.33	0.79 (0.79–0.80)	0.89	< 0.001
Model 6	825.29	0.76 (0.75–0.77)	0.86	< 0.001
Model 7	719.02	0.80 (0.80–0.81)	0.90	

Data in parentheses are 95% CIs. The modeling factors are all independent prognostic factors. Model 1: TNM staging. Model 2: whole emphysema severity. Model 3: ILA severity. Model 4: TNM staging and whole emphysema severity. Model 5: TNM staging and ILA severity. Model 6: whole emphysema severity and ILA severity. Model 7: TNM staging, whole emphysema severity and ILA severity. TNM staging was based on the 9th AJCC system for lung cancer

*p*-value for the comparison of the AUC of model 7 with models 1–6, using DeLong’s test. *p* < 0.05 indicates a statistically significant difference

*TNM* tumor-node-metastasis, *AIC* Akaike information criterion, *AUC* the area under the time-dependent receiver operating characteristic curve, *ILA* interstitial lung abnormality

**Fig. 6:**
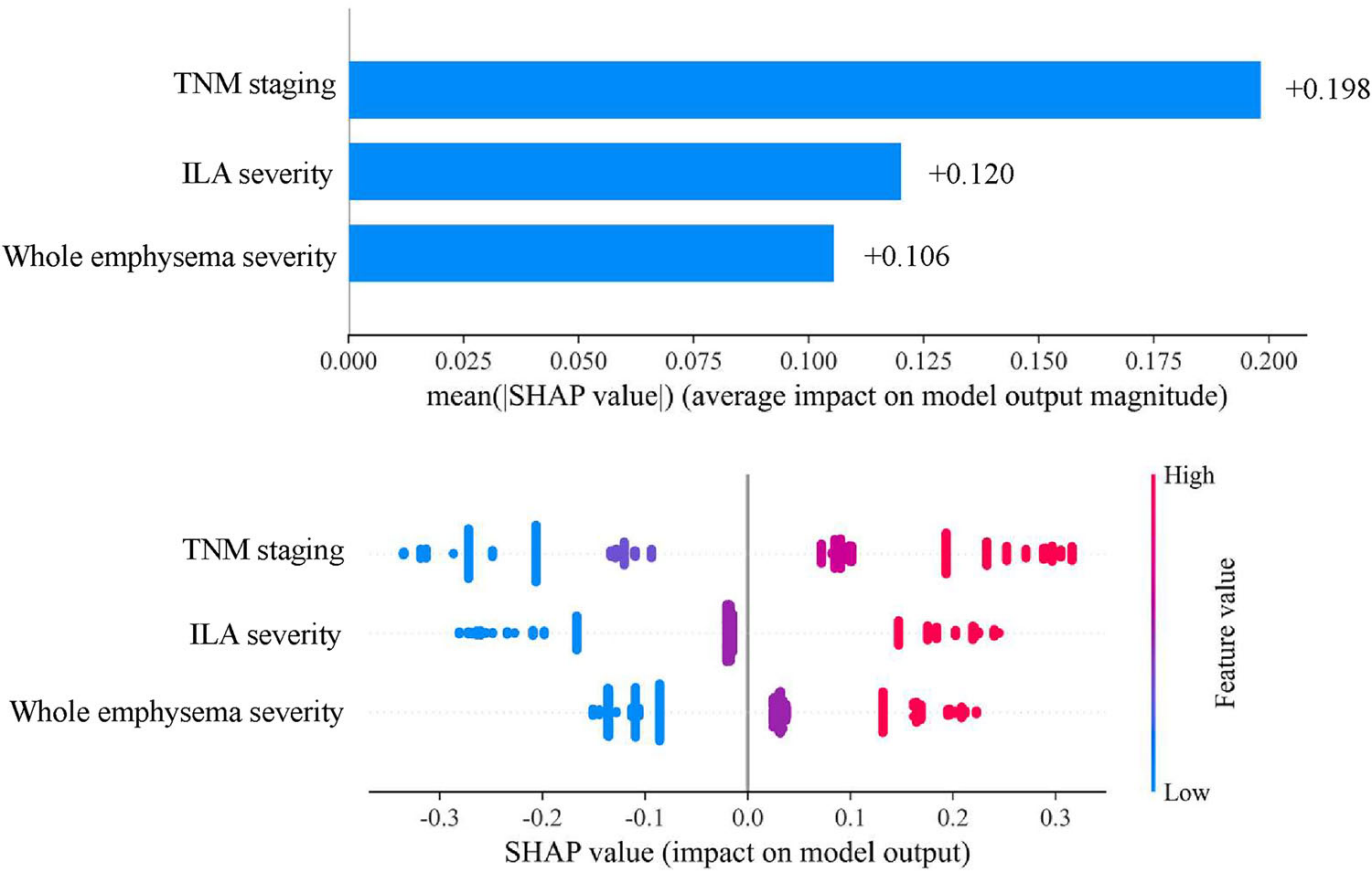
Shapley additive explanation plot visualization for selected predictors. The broader the horizontal bar, the more significant the observed effect. In the color bar on the right side, blue indicates low predictors, and red indicates high predictors. Each feature’s contribution is shown on the *x*-axis. A feature with a positive Shapley value increases the risk of death. Readers can infer the risk of death according to the specific value of each predictor. ILA, interstitial lung abnormality; TNM, tumor-node-metastasis

Using Model 7, which demonstrated the best performance, we generated Shapley additive explanation plots to better visualize the contributions of the factors to the model (Fig. 6). ILA severity was found to be more important than whole emphysema severity in predicting NSCLC patient survival.

### Impact of AI-quantified emphysema and ILA on surgical eligibility in stages I–IIIA

For stage IA patients, mild emphysema (OR, 0.41 [95% CI: 0.32, 0.57]; *p* < 0.001; Table 5), more than mild emphysema (OR, 0.40 [95% CI: 0.28, 0.68]; *p* < 0.001), equivocal ILA (OR 0.51 [95% CI: 0.40, 0.69]; *p* < 0.001) and definite ILA (OR 0.41 [95% CI: 0.31, 0.64]; *p* < 0.001) were independent predictors of surgical resection. Similar results were observed in patients with stages IB–IIIA NSCLC (mild emphysema: OR 0.53, [95% CI: 0.37, 0.87], *p* = 0.002; more than mild emphysema: OR 0.47, [95% CI: 0.31, 0.87], *p* = 0.005; equivocal ILA: OR 0.55, [95% CI: 0.39, 0.83], *p* < 0.001; and definite ILA: OR 0.50, [95% CI: 0.23, 0.72], *p* = 0.007).

**Table 5:** Odds ratio (95% CI and *p*-value) of having received versus not received lung cancer surgical resection by clinicopathological characteristics, CT-defined emphysema severity, and interstitial lung abnormality severity

	Adjusted odds ratio (95% CI) of receiving vs. not receiving lung cancer surgical resection	
Variables	Stage IA	Stage IB–IIIA
Whole emphysema severity (Non-emphysema)		
Mild	0.41 (0.32, 0.57), *p* < 0.001	0.53 (0.37, 0.87), *p* = 0.002
More than mild	0.40 (0.28, 0.68), *p* < 0.001	0.47 (0.31, 0.87), *p* = 0.005
ILA severity (Non-ILA)		
Equivocal ILA	0.51 (0.40, 0.69), *p* < 0.001	0.55 (0.39, 0.83), *p* < 0.001
Definite ILA	0.41 (0.31, 0.64), *p* < 0.001	0.50 (0.23, 0.72), *p* = 0.007

Data in parentheses are 95% CI

Separate analyses were performed for stage IA and stage IB–IIIA. Adjusted for family history, coronary artery calcification, and CEA in stage IA and adjusted for CEA and CA125 in stage IB–IIIA. *p* < 0.05 indicates a statistically significant difference

*CEA* carcinoembryonic antigen, *CA125* carbohydrate antigen 125, *ILA* interstitial lung abnormality

### Stratified analysis of OS by COPD status, treatment type and TNM staging

Patients were divided into three subgroups: (1) non-COPD and COPD; (2) non-surgery and surgery; and (3) stages I–IIIA and stages IIIB–IV (Supplementary Tables S2, 3 and Supplementary Figs. S1–6). The Supplementary Materials provide detailed emphysema and ILA impact on prognosis in different subgroups.

## Discussion

To date, NSCLC prognosis stratification research has primarily focused on TNM staging, while lung cancer comorbidities have been overlooked [4]. In our study, AI-quantified whole emphysema severity and ILA severity were independent prognostic factors, while regional emphysema severity, regional ILA severity and COPD were not. Furthermore, AI-quantified whole emphysema and ILA severity could provide additional prognostic value with respect to TNM staging alone.

The presence of emphysema and ILA by visual assessment was associated with worse OS in NSCLC [13, 35]. However, visual assessment has limitations, including subjectivity and time-consuming, making objective quantification an ideal assistive task for AI [36]. In our study, AI-based assessment of emphysema and ILA showed excellent agreement with experienced radiologists, supporting the feasibility and reliability of AI for objective imaging evaluation. Kaplan–Meier survival curve and multivariable analyses revealed that NSCLC with more than mild emphysema and definite ILA had a higher mortality risk. A higher emphysema grade is associated with higher levels of matrix metalloproteinases and leads to a worse prognosis [37, 38]. Moreover, ILA can represent early pulmonary fibrosis in certain conditions [39]. Patients with definite ILA typically exhibit more severe lung tissue damage and fibrosis than those with equivocal ILA, leading to poorer prognosis [40]. Our study also observed a multiplicative interaction between emphysema and ILA, which has a significant synergistic negative impact on patient survival. Emphysema impairs gas exchange, while ILA reduces diffusing capacity. Their synergistic effects can severely compromise lung function [41]. Furthermore, the opposing physiological effects of emphysema and ILA may obscure functional impairment, delaying diagnosis and treatment, leading to worse outcomes in NSCLC [41]. However, there is no additive interaction between emphysema and ILA. One potential explanation is that changes in DNA methylation and histone modifications, telomere dysfunction and shortening are shared pathogenic mechanisms underlying both emphysema and pulmonary fibrosis [41].

Interestingly, our study found that regional emphysema severity was not an independent prognostic factor in a cohort including patients with stage I–IV, which was inconsistent with previous studies focusing on stages I–II [42]. Furthermore, COPD was also not an independent prognostic factor in our study. Recent studies have shown that some individuals exhibit emphysema and ILA in CT images before being diagnosed with COPD [26, 43]. This indicated that lung function impairment is associated with NSCLC patient outcome regardless of whether it meets the clinical criteria for a COPD diagnosis.

In our study, C-index, AIC and calibration analyses consistently showed that Model 7 outperformed the other models, demonstrating the best goodness-of-fit and robustness. These findings also confirm the added value of AI-quantified emphysema and ILA severity. Therefore, these quantified metrics should be considered in prognosis predictions for NSCLC patients in clinical practice to optimize treatment and monitoring. Moreover, the Shapley additive explanation plots of Model 7 showed that ILA is more important than emphysema in predicting NSCLC survival. This might be because emphysema involves the destruction of alveolar walls, whereas ILA is considered a potentially progressive interstitial lung disease [11, 44]. Interstitial lung disease frequently results in end-stage respiratory failure, particularly pulmonary fibrosis, increase in lung stiffness and the development of scarring [44, 45]. These features indicate that ILA may be related to worse OS than emphysema.

Stages I–IIIA NSCLC patients with emphysema and ILA were less likely to undergo surgery than no emphysema and ILA. Surgical resection remains the standard treatment for stages I–IIIA NSCLC; however, those who also have comorbidities are frequently considered inoperable due to low cardiopulmonary reserve [5, 46]. In clinical practice, patients who are not suitable for surgery may need to consider alternative treatment options, such as stereotactic radiation, targeted therapy and immunotherapy [47]. Consequently, the impact of emphysema and ILA on treatment decisions in patients with stages I–IIIA NSCLC must be fully considered, as this will facilitate the development of individualized treatment plans and improve patients’ prognosis.

In the COPD subgroup, although ILA severity stratified patients by risk in the Kaplan–Meier analysis, it was not an independent prognostic factor. This result is potentially due to shared genome segments, including FAM13A, DSP, and 17q21, between COPD and ILA, where these variants increase the risk of COPD but protect against ILA [44]. Whole emphysema severity was correlated with the mortality rate but was not an independent prognostic factor in patients who underwent surgery. The potential explanation is that patients who underwent surgery had a lower incidence of emphysema; furthermore, postoperative survival is affected by other factors, including age and reduced pulmonary function [48]. Additionally, whole emphysema and ILA severity were independent prognostic factors for NSCLC stage I–IIIA and IIIB–IV patients. Therefore, incorporating AI-quantified emphysema and ILA into the model can optimize the prognostic model’s prediction performance and improve survival risk stratification in NSCLC.

Our study has some limitations. This retrospective single-center study might be subject to potential selection and inherent biases. Therefore, future multicenter prospective clinical studies are needed to confirm our findings. Furthermore, while we investigated the OS outcome of NSCLC patients, the specific cause of mortality was not identified. Our next study will investigate specific causes of mortality in NSCLC patients. Finally, we used X-tile to determine the cutoff value for regional ILA severity since no reference cutoff is available; further validation in diverse populations is necessary.

In conclusion, AI-quantified emphysema and ILA severity not only independently predict increased mortality risk in NSCLC patients but also provide complementary prognostic value to TNM staging. These parameters, derived from baseline CT scans, serve as potential early markers of survival that can help stratify mortality risk in NSCLC patients. Future work will further evaluate the generalizability of these findings.

## Supplementary information

ELECTRONIC SUPPLEMENTARY MATERIAL

## Data Availability

The dataset used or analyzed during the current study is available from the corresponding author on reasonable request.

## Abbreviations

AI: Artificial intelligence
AUC: Area under the receiver operating characteristic curve
CI: Confidence interval
C-index: Concordance index
COPD: Chronic obstructive pulmonary disease
HR: Hazard ratio
ILA: Interstitial lung abnormality
NSCLC: Non-small cell lung cancer
OR: Odds ratio
OS: Overall survival
PFT: Pulmonary function test
ROC: Receiver operating characteristic
TNM: Tumor-node-metastasis
