# Supplementary material for: Association of automated quantified emphysema and interstitial lung abnormality with survival in non-small cell lung cancer
Source: Insights Imaging. 2026 Jan 5;17:7. doi: 10.1186/s13244-025-02180-6 (PMC12770050; doi:10.1186/s13244-025-02180-6)
Supplement: Supplementary file 1 — ELECTRONIC SUPPLEMENTARY MATERIAL [file 13244_2025_2180_MOESM1_ESM.pdf]

Association of Automated Quantified Emphysema and Interstitial Lung Abnormality with Survival in Non-Small Cell Lung Cancer

ELECTRONIC SUPPLEMENTARY MATERIAL

Materials and Methods

Chest CT Scan and Settings Parameters

The following are the scan and settings parameters that were used in the study: tube voltage, 120–130 kV; tube current, 80–160 mAs; rotation time, 0.4–0.6 s; slice gap, 0.5–0.7 mm; pitch, 0.984; matrix size, 512 × 512; and field of view, 330 × 350 mm. The CT images were reconstructed using both a soft tissue kernel (for the emphysema quantification analysis) and a Y sharp kernel (B) (for the interstitial lung abnormality [ILA] quantification analysis), with a slice thickness/interval of 1/1 or 1.25/1.25 mm.

Visual Assessment Criteria of Emphysema and ILA

Two radiologists (T.J.L. and L.C.Y., with 9 and 15 years of thoracic CT experience, respectively) determined whether whole emphysema was present. Subsequently, patients with emphysema were graded as having trace, mild, moderate, confluent, or advanced destructive emphysema based on the Fleischner Society's visual classification criteria [1]. Additionally, the radiologists assessed the presence of ILA, and ILA patients were categorized into two severities: (1) equivocal ILA (the presence of any nondependent changes affected less than 5% of any lung zone or with unilateral changes); and (2) definite ILA (affected more than or equal to 5% of any lung zone) [2]. Radiologists assessed the CT imaging together and reached an agreement on the presence of whole emphysema and its severity, as well as the presence of ILA and its severity. During the visual assessments, radiologists were blinded to all patients' clinicopathologic data and the artificial intelligence (AI) software results.

Subgroup Analysis

The survival analysis of subgroups was described, and it included the Kaplan-Meier curves and the univariable and multivariable Cox regression proportional hazards model. Different predictive models were then established according to the result of the subgroup multivariable Cox regression analyses. The model performances were then evaluated using time-dependent receiver operating characteristic curves, concordance index (C-index), calibration curves, and decision curves.

The Variables Analysis, the Agreement and Correlation Analysis Between the Observer and AI

The distribution of continuous variables was assessed using the Kolmogorov-Smirvov test. All continuous variables had non-normal distributions; hence, they were reported as the median and interquartile range. The Mann–Whitney U test or Kruskal–Wallis test was applied to analyze the continuous data. Categorical variables are shown as the frequency and percentage, and the Pearson χ^2 or Fisher exact test were used to compare the categorical variables. The agreement and correlation between the observer and AI were assessed using the weighted κ coefficient and Spearman correlation coefficients, respectively. The κ values were graded as follows: poor, 0–0.20; fair, 0.21–0.40; moderate, 0.41–0.60; good, 0.61–0.80; and excellent, 0.81–1.00.

Results

Performance of Models 1 - 6

Model 1 displayed a better predictive strength that of Model 2 and Model 3 (C-index: 0.76 vs 0.71 and 0.71; 5-year areas under the receiver operating characteristic curve (AUC): 0.85 vs 0.79 and 0.79). Compared to Model 5, Model 4 and Model 6 had poorer efficiency in predicting NSCLC outcome (C-index: 0.79 vs 0.78 and 0.76; 5-year AUC: 0.89 vs 0.88 and 0.86).

Emphysema and ILA Impact on Prognosis in Different Subgroups

Non-COPD and COPD groups. –The Kaplan–Meier analysis demonstrated that patients without whole emphysema, regional emphysema, ILA and regional ILA had higher 5-year OS rates than patients with emphysema and ILA both in the COPD and the non-COPD groups. There was no statistical difference in the prognosis between the no regional ILA and low regional ILA in the COPD group (p =0.76; Supplementary Figure S1). The multivariable Cox regression analysis indicated that the whole emphysema severity (mild: hazard ratio [HR], 1.53 [95% CI: 1.09, 2.16]; p =0.01; and more than mild: HR, 2.60 [95% CI: 1.73, 3.90; p <0.001) and ILA severity (equivocal ILA: HR, 1.62 [95% CI: 1.05, 2.50]; p =0.03; and definite ILA: HR, 2.83 [95% CI: 1.77, 4.50]; p <0.001) were independent prognostic factors in the non-COPD group (Supplementary Table S2). In the COPD group, the whole emphysema severity (mild: HR, 1.73 [95% CI: 1.07, 2.79]; p =0.03; and more than mild: HR, 2.40 [95% CI: 1.45, 3.97; p <0.001) was identified as an independent prognostic factor, and the ILA severity was not. Model $_{\text{TNM staging + whole emphysema severity + ILA severity}}$ (C-index: 0.82; 5-year AUC, 0.89) had best predictive performance in the non-COPD group and Model $_{\text{TNM staging + whole emphysema severity}}$ (C-index: 0.70; 5-year AUC: 0.88) displayed an outstanding performance in the COPD group (Supplementary Table S3 and Supplementary Figure S2A).

Non-surgery and Surgery groups. –The Kaplan–Meier analysis, showed that patients without whole emphysema, regional emphysema, ILA and regional ILA had better outcome compared to those with emphysema and ILA (Supplementary Figure S3). In the non-surgery group, the whole emphysema severity (mild: HR, 2.02 [95% CI: 1.43, 2.85]; p <0.001; and more than mild: HR, 3.28 [95% CI: 2.24, 4.81; p <0.001) and ILA severity (definite ILA: HR, 1.75 [95% CI: 1.03, 2.96]; p =0.04) were independent prognostic factors (Supplementary Table S2). Surgery patients with equivocal ILA (HR, 2.35 [95% CI: 1.36, 4.09]; p =0.002) and definite ILA (HR, 5.91 [95% CI: 3.24, 10.78]; p <0.001) were at greater risk of mortality than patients with non-ILA, while whole emphysema severity did not show this statistical association.

Model $_{\text{TNM staging + whole emphysema severity + ILA severity}}$ (C-index: 0.76; 5-year AUC, 0.90) had optimal predictive performance in the non-surgery group, while in the surgery group was Model $_{\text{TNM staging + ILA severity}}$ (C-index: 0.82; 5-year AUC, 0.89) (Supplementary Table S3 and Supplementary Figure S4A).

Stages I-IIIA and Stages IIIB-IV groups. –The Kaplan–Meier analysis showed that the severity of whole emphysema, regional emphysema, ILA and regional ILA increased while the patient survival rates decreased both in the stages I-IIIA group and the stages IIIB-IV group (Supplementary Figure S5). Moreover, the multivariable Cox regression analysis indicated that the whole emphysema severity and ILA severity were independent prognostic factors in both groups (Supplementary Table S2). In the stages I-IIIA group and stages IIIB-IV group, the C-index of the Model $_{\text{whole emphysema severity + ILA severity}}$ were 0.77 and 0.61, and the 5-year AUC were 0.81 and 0.79, respectively (Supplementary Table S3 and Supplementary Figure S6A).

The regional emphysema severity and regional ILA severity were still not independent prognostic factors in all subgroups. Additionally, the calibration curve of the best-performing model fit well (Supplementary Figure S2B, Supplementary Figure S4B and Supplementary Figure S6B), and its decision curve analysis showed a good net benefit (Supplementary Figure S2C, Supplementary Figure S4C and Supplementary Figure S6C).

Acknowledgments

We thank LetPub (www.letpub.com.cn) for its linguistic assistance during the preparation of this manuscript.

References

1 Lynch DA, Moore CM, Wilson C et al (2018) CT-based Visual Classification of Emphysema: Association with Mortality in the COPDGene Study. Radiology 288:859-866

2 Lee JE, Chae KJ, Suh YJ et al (2023) Prevalence and Long-term Outcomes of CT Interstitial Lung Abnormalities in a Health Screening Cohort. Radiology 306:e221172

Supplementary Table S1: Association between Regional ILA Severity and Overall Survival in Sensitivity Analyses

	Excluding participants whose death occurred within the first year of follow-up (n = 1586)		Age (< 65 years)		Age (≥65 years)		Male		Female	
**Variables**	**HR (95% CI)**	***p* value**	**HR (95% CI)**	***p* value**	**HR (95% CI)**	***p* value**	**HR (95% CI)**	***p* value**	**HR (95% CI)**	***p* value**
Regional ILA severity (No)										
Low	0.98 (0.70, 1.36)	0.89	0.97 (0.63, 1.50)	0.90	0.84 (0.52, 1.36)	0.49	0.70 (0.43, 1.13)	0.14	1.25 (0.80, 1.94)	0.33
High	1.32 (0.95, 1.84)	0.10	1.32 (0.85, 2.04)	0.22	1.21 (0.75, 1.94)	0.44	0.96 (0.60, 1.54)	0.90	1.83 (0.93, 1.89)	0.10

	Using the multiple imputation on Variables (n = 1720)		Smoking history (Yes)		Smoking history (No)		Tumor size (< 3cm)		Tumor size (≥ 3cm)	
**Variables**	**HR (95% CI)**	***p* value**	**HR (95% CI)**	***p* value**	**HR (95% CI)**	***p* value**	**HR (95% CI)**	***p* value**	**HR (95% CI)**	***p* value**
Regional ILA severity (No)										
Low	0.95 (0.70, 1.30)	0.76	0.94 (0.72, 1.31)	0.16	1.45 (0.96, 2.19)	0.08	1.01 (0.62, 1.64)	0.98	0.93 (0.60, 1.44)	0.73
High	1.31 (0.96, 1.79)	0.09	0.78 (0.48, 1.28)	0.33	1.54 (0.83, 2.61)	0.31	1.52 (0.91, 2.54)	0.11	1.20 (0.78, 1.84)	0.41

Note.—Hazard ratios (HRs) and 95% confidence intervals (95%CIs) were calculated using Cox proportional hazards models. CEA =

carcinoembryonic antigen; CA125 = carbohydrate antigen 125; TNM = tumor-node-metastasis; COPD = chronic obstructive pulmonary disease; ILA = interstitial lung abnormality. p <0.05 indicates a statistically significant difference.

Adjusted for age, sex, smoking history, family history, hypertension, CEA, CA125, tumor size, TNM staging, treatment, histologic type, tumor location, COPD status, whole emphysema severity, regional emphysema severity, and ILA severity in the model (excluding participants whose death occurred within the first year of follow-up).

Adjusted for sex, smoking history, family history, CEA, CA125, tumor size, TNM staging, treatment, histologic type, tumor location, COPD status, whole emphysema severity, regional emphysema severity, and ILA severity in the age (< 65 years) model. Adjusted for CEA, CA125, tumor size, TNM staging, treatment, Tumor location, COPD status, whole emphysema severity, regional emphysema severity, and ILA severity in the age (≥ 65 years) model.

Adjusted for age, smoking history, CEA, CA125, tumor size, TNM staging, treatment, histologic type, tumor location, COPD status, whole emphysema severity, regional emphysema severity, and ILA severity in the sex (male) model. Adjusted for age, family history, coronary artery calcification, CEA, CA125, tumor size, TNM staging, treatment, histologic type, tumor location, COPD status, whole emphysema severity, regional emphysema severity, and ILA severity in the sex (female) model.

Adjusted for age, diabetes mellitus, coronary artery calcification, CEA, CA125, tumor size, TNM staging, treatment, histologic type, tumor location, COPD status, whole emphysema severity, regional emphysema severity, and ILA severity in the smoking history (yes) model. Adjusted for age, sex, family history, hypertension, coronary artery calcification, CEA, CA125, tumor size, TNM staging, treatment, histologic type, tumor location, COPD status, whole emphysema severity, regional emphysema severity, and ILA severity in the smoking history (no) model.

Adjusted for age, sex, smoking history, family history, hypertension, coronary artery calcification, CEA, CA125, TNM staging, treatment, histologic type, tumor location, COPD status, whole emphysema severity, regional emphysema severity, and ILA severity in the tumor size (< 3 cm) model. Adjusted for age, CEA, CA125, TNM staging, treatment, tumor location, COPD status, whole emphysema severity, regional emphysema severity, and ILA severity in the tumor size (≥ 3 cm) model.

Adjusted for age, sex, smoking history, family history, hypertension, CEA, CA125, tumor size, TNM staging, treatment, histologic type, tumor location, COPD status, whole emphysema severity, regional emphysema severity, and ILA severity in the model (using the multiple imputation on variables).

Supplementary Table S2: Association between the AI-Quantified Metrics and Overall Survival in Different Subgroups

Variables	COPD Status		Treatment Type		TNM Staging	
	**Non-COPD (n = 1173)**	**COPD (n = 502)**	**Non-surgery (n = 932)**	**Surgery (n = 743)**	**Stages I-IIIA (n = 839)**	**Stages IIIB-IV (n = 836)**
Whole emphysema severity (Non-emphysema)						
Mild	1.53 (1.09, 2.16), *p* = 0.01	1.73 (1.07, 2.79), *p* = 0.03	2.02 (1.43, 2.85), *p* < 0.001	1.07 (0.64, 1.77), *p* = 0.81	2.39 (1.43, 3.97), *p* < 0.001	1.43 (1.04, 1.98), *p* = 0.03
More than mild	2.60 (1.73, 3.90), *p* < 0.001	2.40 (1.45, 3.97), *p* < 0.001	3.28 (2.24, 4.81), *p* < 0.001	1.54 (0.84, 2.82), *p* = 0.16	2.60 (1.26, 5.40), *p* = 0.01	2.28 (1.61, 3.22), *p* < 0.001
Regional emphysema severity (No)						
Low	1.23 (0.87, 1.74), *p* = 0.24	0.80 (0.49, 1.29), *p* = 0.35	0.92 (0.64, 1.33), *p* = 0.51	1.05 (0.68, 1.63), *p* = 0.83	1.17 (0.74, 1.85), *p* = 0.51	0.99 (0.71, 1.39), *p* = 0.97
High	0.95 (0.61, 1.47), *p* = 0.81	0.61 (0.36, 1.03), *p* = 0.06	1.20 (0.43, 1.98), *p* = 0.12	0.72 (0.40, 1.31), *p* = 0.29	1.44 (0.69, 3.02), *p* = 0.33	0.68 (0.47, 1.01), *p* = 0.05
ILA severity (Non-ILA)						
Equivocal ILA	1.62 (1.05, 2.50), *p* = 0.03	1.55 (0.82, 2.9), *p* = 0.18	1.38 (0.83, 2.31), *p* = 0.22	2.35 (1.36, 4.09), *p* = 0.002	3.18 (1.61, 6.30), *p* < 0.001	1.35 (0.88, 2.08), *p* = 0.17
Definite ILA	2.83 (1.77, 4.50), *p* < 0.001	1.87 (0.99, 3.50), *p* = 0.05	1.75 (1.03, 2.96), *p* = 0.04	5.91 (3.24, 10.78), *p* < 0.001	5.66 (2.56, 12.50), *p* <0.001	2.05 (1.33, 3.16), *p* = 0.001
Regional ILA severity (No)						
Low	1.03 (0.69, 1.55), *p* = 0.88	0.79 (0.46, 1.37), *p* = 0.40	0.84 (0.52, 1.37), *p* = 0.50	1.23 (0.77, 1.98), *p* = 0.39	1.06 (0.59, 1.91), *p* = 0.85	0.90 (0.61, 1.33), *p* = 0.60

High	1.48 (0.97, 2.26), p = 0.07	1.05 (0.63, 1.76), p = 0.86	1.22 (0.75, 1.98), p = 0.41	1.56 (0.97, 2.52), p = 0.07	1.72 (0.93, 3.19), p = 0.08	1.20 (0.82, 1.78), p = 0.35

Note. Hazard ratios (HRs) and 95% confidence intervals (95% CIs) were calculated using Cox proportional hazards models. CEA = carcinoembryonic antigen; CA125 = carbohydrate antigen 125; TNM = tumor-node-metastasis; COPD = chronic obstructive pulmonary disease; and ILA = interstitial lung abnormality. $p < 0.05$ indicates a statistically significant difference.

Adjusted for age, sex, smoking history, hypertension, coronary artery calcification, CEA, CA125, tumor size, TNM staging, treatment, histologic type, and the tumor location in the COPD status (Non-COPD) model. Adjusted for family history, CEA, CA125, tumor size, TNM staging, and treatment in the COPD status (COPD) model.

Adjusted for age, sex, smoking history, hypertension, CEA, CA125, tumor size, TNM staging, COPD status, histologic type, and tumor location in the treatment type (Non-surgery) model. Adjusted for age, family history, CEA, CA125, tumor size, TNM staging, COPD status, histologic type, and the tumor location in the treatment type (surgery) model.

Adjusted for age, CEA, CA125, tumor size, treatment, histologic type, tumor location, and COPD status in the TNM staging (Stages I-IIIA) model. Adjusted for age, sex, smoking history, family history, CEA, CA125, tumor size, treatment, tumor location, COPD status, and histologic type in the TNM staging (Stages IIIB-IV) model.

Supplementary Table S3: Additional Predictive Value of AI-quantified Emphysema and ILA in Different Subgroups

	Non-COPD group			COPD group			Non-surgery group			Surgery group			Stages I-IIIa group		
	C-Index	5-year AUC	*p* value	C-Index	5-year AUC	*p* value	C-Index	5-year AUC	*p* value	C-Index	5-year AUC	*p* value	C-Index	5-year AUC	*p* value
Model $_{\text{TNM staging}}$	0.76	0.82	<0.001	0.68	0.84	<0.001	0.71	0.84	<0.001	0.77	0.81	<0.001			
Model $_{\text{whole emphysema severity}}$	0.72	0.78	<0.001	0.62	0.77	<0.001	0.68	0.79	<0.001				0.68	0.71	<0.001
Model $_{\text{ILA severity}}$	0.73	0.79	<0.001				0.65	0.76	<0.001	0.76	0.81	<0.001	0.72	0.75	<0.001
Model $_{\text{TNM staging + whole emphysema severity}}$	0.79	0.86	<0.001	0.70	0.88		0.75	0.88	<0.001						
Model $_{\text{TNM staging + ILA severity}}$	0.81	0.88	<0.001				0.74	0.87	<0.001	0.82	0.89				
Model $_{\text{whole emphysema severity + ILA severity}}$	0.78	0.86	<0.001				0.71	0.84	<0.001				0.77	0.81	
Model $_{\text{TNM staging + whole emphysema severity + ILA severity}}$	0.82	0.89					0.76	0.9							

Note. The modeling factors are all independent prognostic factors. The model with the highest AUC value was compared to other models using Delong's test to assess the performances in different subgroups. AUC = the area under the time-dependent receiver operating characteristic curve; C-index = concordance index; TNM = tumor-node-metastasis; COPD = chronic obstructive pulmonary disease; and ILA = interstitial lung abnormality. A $p < 0.05$ indicates a statistically significant difference.

Supplementary Figures

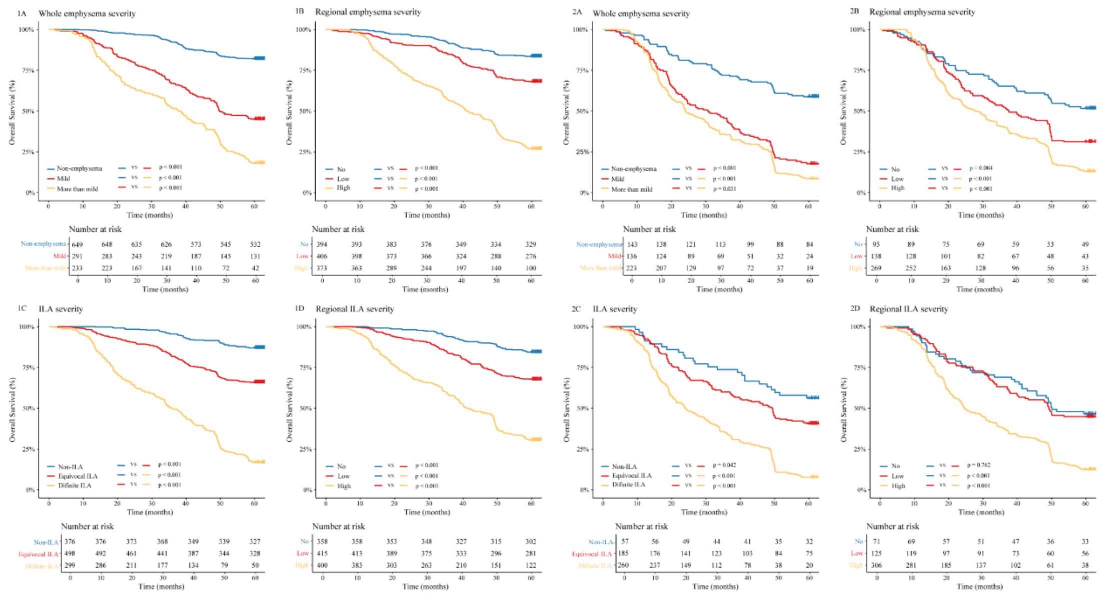

Supplementary Figure S1: Kaplan–Meier curves showing the 5-year overall survival rate in the non-COPD group (1A-1D) and COPD group (2A-2D).

Note: Whole emphysema severity (A), regional emphysema severity (B), ILA severity (C), and regional ILA severity (D). COPD = chronic obstructive pulmonary disease; ILA= interstitial lung abnormality. $p < 0.05$ was statistically significant.

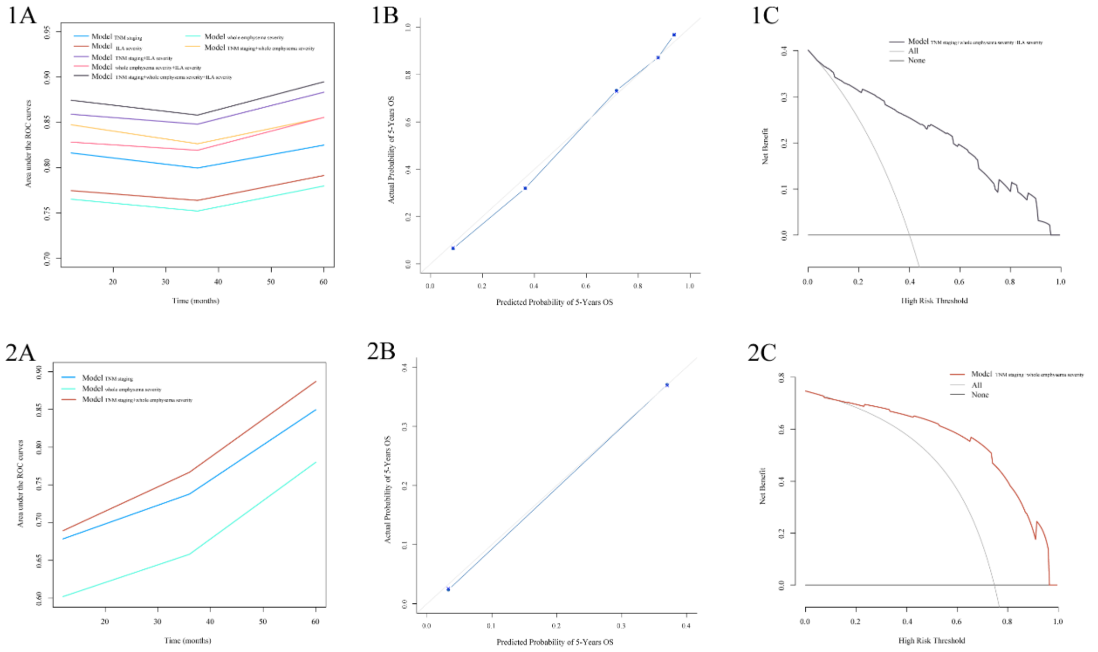

Supplementary Figure S2: Prognostic performances and clinical usefulness of models in the non-COPD group and COPD group.

Note: (A) Time-dependent areas under the ROC curve of models. (B) The calibration curve of model $_{\text{TNM staging + whole emphysema severity + ILA severity}}$ (non-COPD group) and model $_{\text{TNM staging + whole emphysema severity}}$ (COPD group) shows the agreement between the predicted and observed 5-year survival rates. The Hosmer–Lemeshow goodness-of-fit test showed that the *p*-values for the models were greater than 0.05. (C) The decision curve analysis of model $_{\text{TNM staging + whole emphysema severity + ILA severity}}$ (non-COPD group) and model $_{\text{TNM staging + whole emphysema severity}}$ (COPD group). The y-axis measures the net benefit, while the x-axis represents the different probability thresholds of the mortality risk. The gray line represents the assumption that all patients died by the fifth year. The black line represents the assumption that no patients experienced death by the fifth year. The modeling factors were all independent prognostic factors in the non-COPD group and COPD group. ROC = the time-dependent receiver operating characteristic curve; COPD = chronic obstructive pulmonary disease; ILA = interstitial lung abnormality; and TNM = tumor-node-metastasis.

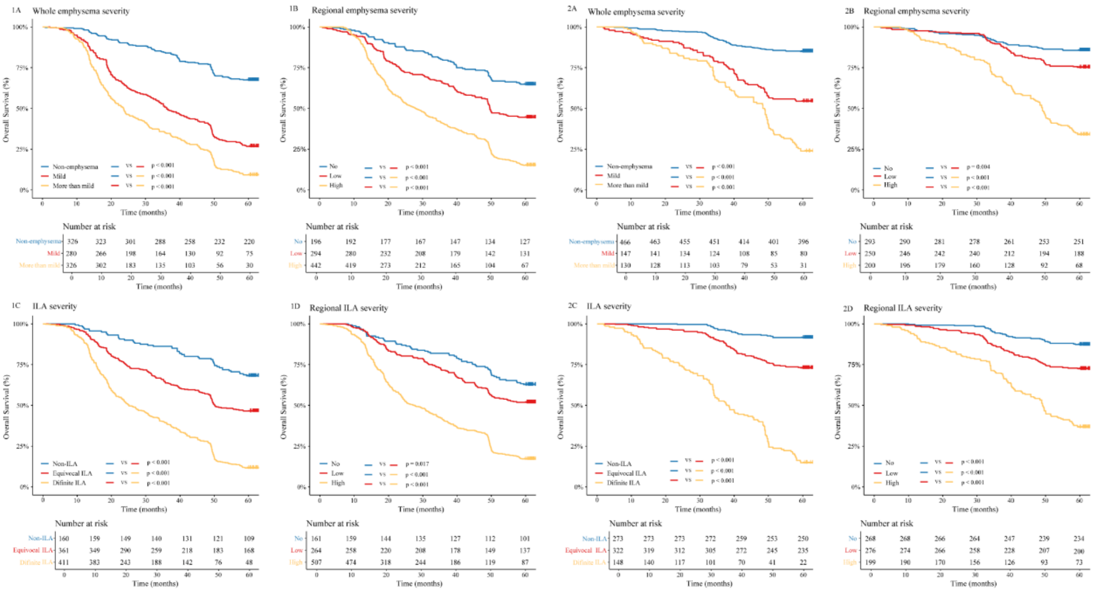

Supplementary Figure S3: Kaplan–Meier curves showing the 5-year overall survival rate in the non-surgery group (1A-1D) and surgery group (2A-2D).

Note: Whole emphysema severity (A), regional emphysema severity (B), ILA severity (C), and regional ILA severity (D). ILA= interstitial lung abnormality. p <0.05 was statistically significant.

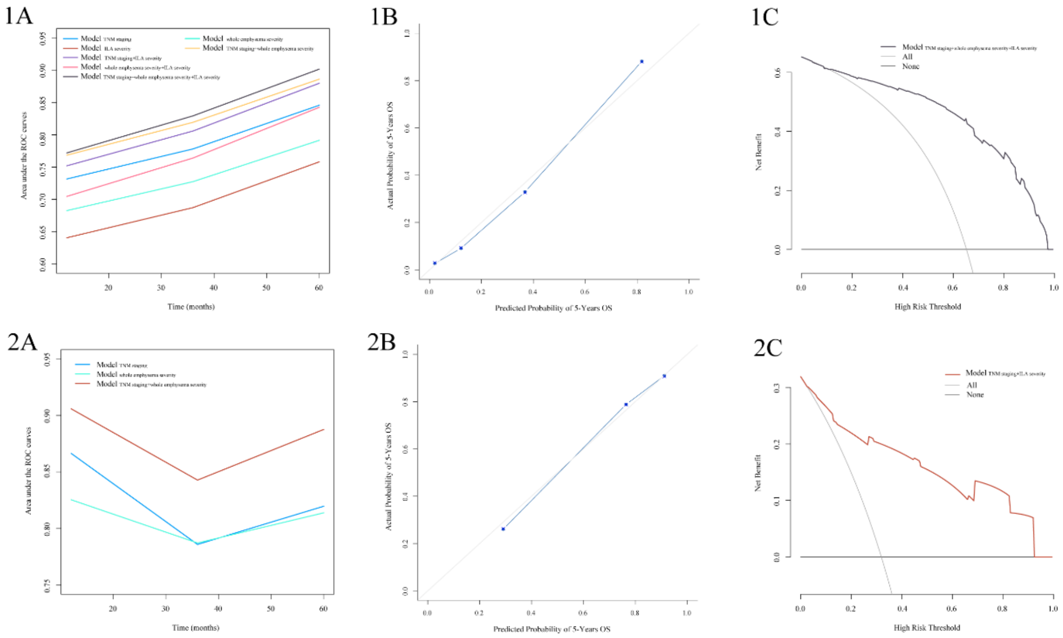

Supplementary Figure S4: Prognostic performance and clinical usefulness of models in the non-surgery group and the surgery group.

Note: (A) Time-dependent areas under the ROC curve of the models. (B) The calibration curve of model $_{\text{TNM staging + whole emphysema severity + ILA severity}}$ (non-surgery group) and model $_{\text{TNM staging + ILA severity}}$ (surgery group) shows the agreement between the predicted and observed 5-year survival rates. The Hosmer–Lemeshow goodness-of-fit test shows that the *p*-values for the models were greater than 0.05. (C) The decision curve analysis of model $_{\text{TNM staging + whole emphysema severity + ILA severity}}$ (non-surgery group) and model $_{\text{TNM staging + ILA severity}}$ (surgery group). The y-axis measures the net benefit, while the x-axis represents the different probability thresholds of the mortality risk. The gray line represents the assumption that all patients died by the fifth year. The black line represents the assumption that no patients experienced death by the fifth year. The modeling factors are all independent prognostic factors in the non-surgery group and surgery group. ROC = the time-dependent receiver operating characteristic curve; ILA = interstitial lung abnormality; and TNM = tumor-node-metastasis.

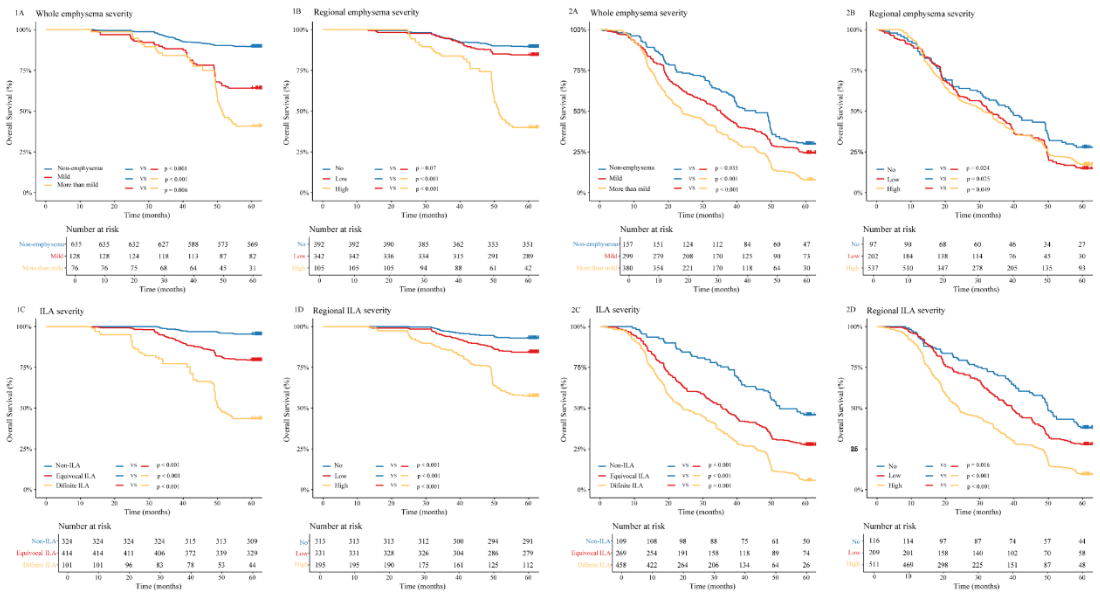

Supplementary Figure S5: **Kaplan–Meier curves show 5-year overall survival rate in stages I-IIIA group (1A-1D) and stages IIIB-IV group (2A-2D)**.

Note: Whole emphysema severity (A), regional emphysema severity (B), ILA severity (C), and regional ILA severity (D). ILA= interstitial lung abnormality. p <0.05 was statistically significant.

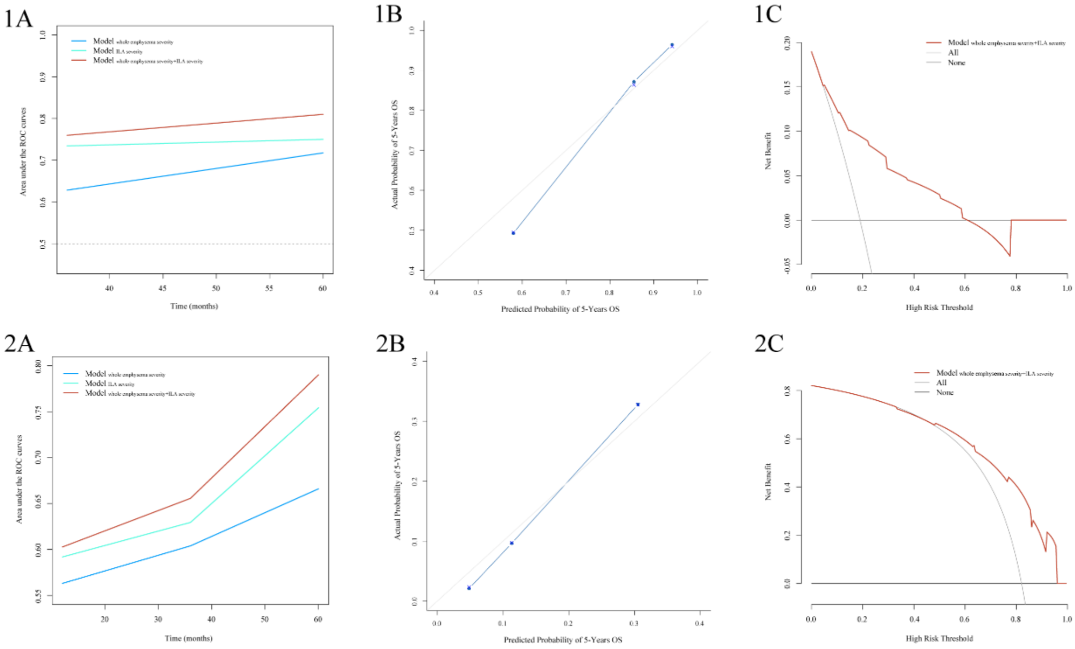

Supplementary Figure S6: Prognostic performance and clinical usefulness of the models in the TNM stages I-IIIA group and TNM stages IIIB-IV group.

Note: (A) Time-dependent areas under the ROC curve of the models. (B) The calibration curve of model $_{\text{whole emphysema severity + ILA severity}}$ (TNM stages I-IIIA group and TNM stages IIIB-IV group) shows the agreement between the predicted and observed 5-year survival rates. The Hosmer–Lemeshow goodness-of-fit test shows that the *p*-values for the models were greater than 0.05. (C) The decision curve analysis of model $_{\text{whole emphysema severity + ILA severity}}$ (TNM stages I-IIIA group and TNM stages IIIB-IV group). The y-axis measures the net benefit, while the x-axis represents the different probability thresholds of the mortality risk. The gray line represents the assumption that all patients died by the fifth year. The black line represents the assumption that no patients experienced death by the fifth year. The modeling factors were all independent prognostic factors in the stage I-IIIa group and stages IIIB-IV group. ROC = the time-dependent receiver operating characteristic curve; ILA = interstitial lung abnormality; and TNM = tumor-node-metastasis.
